# Specialized endoplasmic reticulum‐derived vesicles in plants: Functional diversity, evolution, and biotechnological exploitation

**DOI:** 10.1111/jipb.13233

**Published:** 2022-03-09

**Authors:** Xie Li, Xifeng Li, Baofang Fan, Cheng Zhu, Zhixiang Chen

**Affiliations:** ^1^ College of Life Science, Key Laboratory of Marine Food Quality and Hazard Controlling Technology of Zhejiang Province China Jiliang University Hangzhou 310018 China; ^2^ Department of Botany and Plant Pathology, Center for Plant Biology Purdue University West Lafayette 47907‐2054 IN USA

**Keywords:** endoplasmic reticulum‐derived vesicles, ER body, glucosinolates, myrosinases, precursor accumulating vesicles, precursor protease vesicles, prolamins, protein body, ricinosomes, zeins

## Abstract

A central role of the endoplasmic reticulum (ER) is the synthesis, folding and quality control of secretory proteins. Secretory proteins usually exit the ER to enter the Golgi apparatus in coat protein complex II (COPII)‐coated vesicles before transport to different subcellular destinations. However, in plants there are specialized ER‐derived vesicles (ERDVs) that carry specific proteins but, unlike COPII vesicles, can exist as independent organelles or travel to the vacuole in a Golgi‐independent manner. These specialized ERDVs include protein bodies and precursor‐accumulating vesicles that accumulate storage proteins in the endosperm during seed development. Specialized ERDVs also include precursor protease vesicles that accumulate amino acid sequence KDEL‐tailed cysteine proteases and ER bodies in Brassicales plants that accumulate myrosinases that hydrolyzes glucosinolates. These functionally specialized ERDVs act not only as storage organelles but also as platforms for signal‐triggered processing, activation and deployment of specific proteins with important roles in plant growth, development and adaptive responses. Some specialized ERDVs have also been exploited to increase production of recombinant proteins and metabolites. Here we discuss our current understanding of the functional diversity, evolutionary mechanisms and biotechnological application of specialized ERDVs, which are associated with some of the highly remarkable characteristics important to plants.

## Graphical abstract

Plants produce specialized endoplasmic reticulum (ER)‐derived vesicles (ERDVs) that carry specific ER‐synthesized proteins but do not travel through the classic ER‐to‐Golgi secretory pathway. These ERDVs are directly associated with important plant traits. This review discusses our current understanding of the functional diversity, evolution and biotechnological uses of ERDVs in plants.

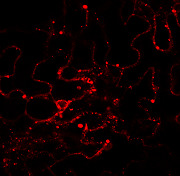

## INTRODUCTION

**Figure 1:**
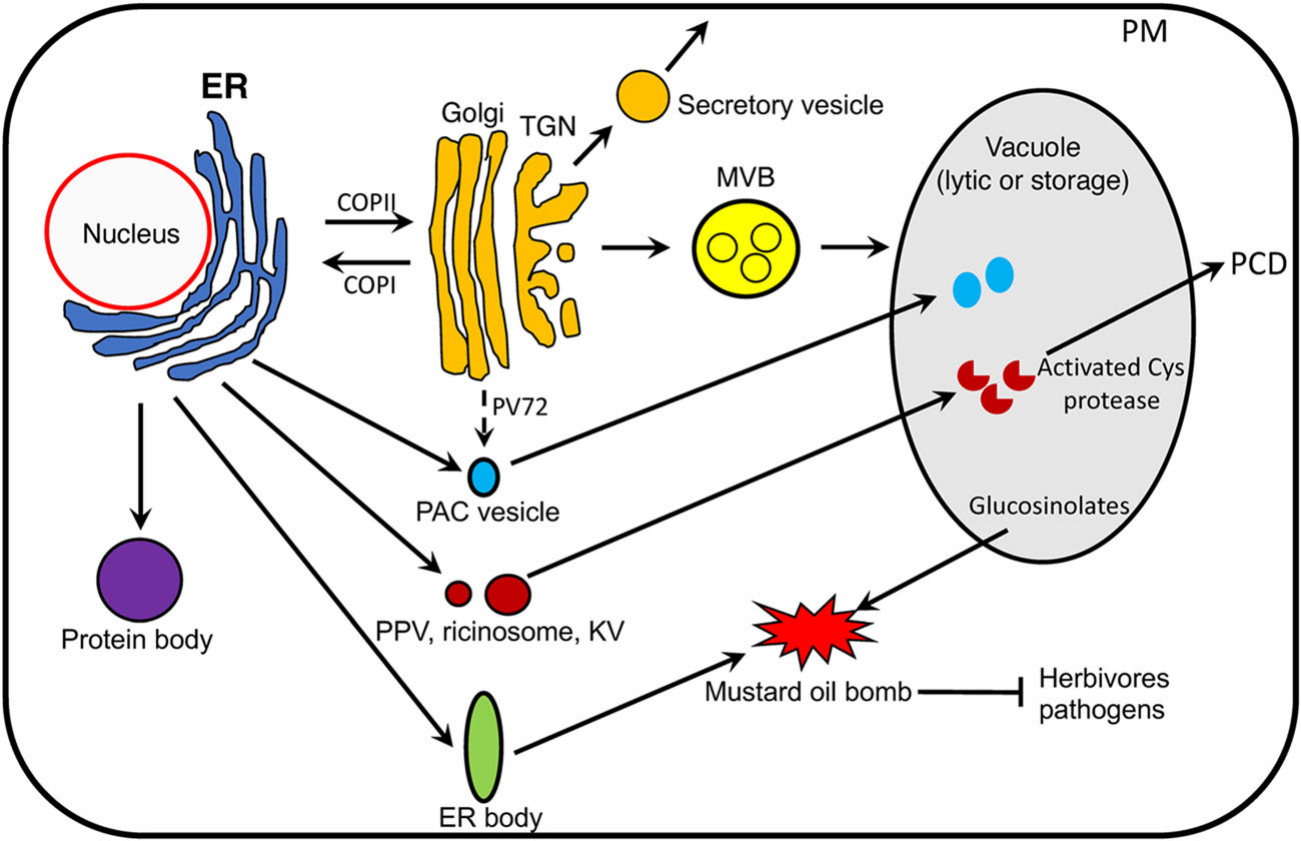
A schematic diagram of different trafficking routes of endoplasmic reticulum (ER)‐synthesized proteins ER proteins can be transported to the vacuole, plasma membrane or extracellular space through the conserved ER‐to‐Golgi secretory pathway. Some storage proteins such as prolamins from cereal plants can form protein bodies as an independent organelle in maturing seeds. Precursors of other storage proteins form aggregates upon synthesis on the ER and develop into precursor‐accumulating (PAC) vesicles for direct trafficking into the protein storage vacuole for further processing in a Golgi‐independent manner. Some of these storage protein precursors may leave the ER for the Golgi apparatus but are recruited back to the PAC vesicles through the action of the vacuolar sorting receptor PV72. Upon synthesis in the ER, amino acid sequence KDEL‐tailed Cys proteases are stored in specialized ER‐derived vesicles (ERDVs) variously known as precursor protease vesicles (PPVs), ricinosomes and KDEL‐tailed Cys protease‐accumulating vesicles (KVs) as inactive proenzymes but are activated into mature enzymes after transport to the vacuole to promote programmed cell death (PCD). ER bodies from Brassicales accumulate a family of β‐glucosidases with a myrosinase activity that can get access to glucosinolates from the vacuole upon tissue damage to produce toxic compounds as mustard bombs against herbivores and pathogens. PM, plasma membrane; MVB, multivesicular body; TGN, trans‐Golgi network.

The endoplasmic reticulum (ER) constitutes an extensive and highly dynamic network of interconnected tubules and cisternae distributed throughout the cell. The ER contains structurally distinct domains including the nuclear envelope, ribosome‐associated rough ER, ribosome‐free smooth ER and the contact regions with other organelles (Schwarz and Blower, 2016). The ER is an important organelle for lipid and protein synthesis and for calcium (Ca^2+^) storage. The highly conserved protein secretion pathway starts at the ER, where secretory proteins are synthesized, fold and pass onto the Golgi apparatus through coat protein complex II (COPII)‐coated vesicles before transport to other endomembrane compartments and extracellular space (Benham, 2012; Adams et al., 2019). As sessile organisms, plant cells highly regulate their endomembrane system, particularly the ER, which is known to be highly flexible and adaptable (Stefano and Brandizzi, 2018). In addition, plants have various types of functionally specialized ER‐derived vesicles (ERDVs) (Chrispeels and Herman, 2000; Gietl and Schmid, 2001; Matsushima et al., 2003a; Takahashi et al., 2005; Yamada et al., 2009; Cheung et al., 2021). Unlike COPII vesicles, these specialized ERDVs carry specific cargo molecules but do not travel through the well‐characterized ER‐to‐Golgi transport pathway. Based on their contents, plant‐specialized ERDVs can be divided into two classes: storage proteins and hydrolytic enzymes. Storage protein ERDVs include protein bodies and precursor‐accumulating vesicles (PACs) in storage organs of cereal and pumpkin seeds, respectively (Figure 1). Protein bodies can exist as independent storage organelles or traffic specific storage proteins directly from the ER to the storage vacuole without passing through the Golgi apparatus (Chrispeels and Herman, 2000; Hara‐Nishimura et al., 2004). Hydrolytic ERDVs include precursor protease vesicles (PPVs) and ricinosomes in the storage tissues of legume seedlings that accumulate amino acid sequence KDEL‐ER‐retention signal‐tailed cysteine (Cys) proteases (Schmid et al., 1998; Okamoto et al., 2003) (Figure 1). ER bodies are another type of hydrolytic ERDVs produced only by plants in the Brassicales order that carry a family of β‐glucosidases with a myrosinase activity for hydrolyzing glucosinolates (Yamada et al., 2020) (Figure 1). These specialized ERDVs function not only as organelles for processing and storage of seed proteins, but also as a platform for signal‐triggered activation, release and deployment of specific cargo proteins important for rapid and timely execution of programmed cell death (PCD) and defense responses. Some of these specialized ERDVs such as protein bodies and ER bodies are present only in some plants and are associated with special traits, providing an excellent system for analyzing the evolutionary basis of plant phenotypic variation. Plant ERDVs have also been exploited for production of recombinant proteins and metabolic engineering (Saberianfar et al., 2016; Saberianfar and Menassa, 2017; Reifenrath et al., 2020). In this review, we discuss what we currently know, what questions remain and how a better knowledge about the diversity, function, evolution and biogenesis of specialized ERDVs can help understand the molecular and cellular basis of important and diverse functional traits in plants. The ER, COPII machinery and COPII vesicle also contribute to autophagosome biogenesis, and autophagy targets ER degradation during ER stress and mediates trafficking of proteins from the ER directly to the vacuole (Liu et al., 2012; Liu and Bassham, 2013; Le Bars et al., 2014; Yang et al., 2016; Zhou et al., 2018; Zhuang et al., 2018; Michaeli et al., 2019; Stephani et al., 2020; Zeng et al., 2021). These subjects will not be discussed here because they have been extensively reviewed (Michaeli et al., 2014; Soto‐Burgos et al., 2018; Zhuang et al., 2018; Bao and Bassham, 2020).

## FUNCTIONAL DIVERSITY OF SPECIALIED ERDVS

### Storage protein ERDVs: Protein bodies and precursor accumulating vesicles

Storage proteins serve important functions throughout the life cycle of plants from seed gemination to growth of vegetative tissue to seed setting for reproduction. Storage proteins in seeds and vegetative tissues are also the major ingredients of food consumed by humans and livestock. All storage proteins are synthesized on the rough ER and can accumulate in protein bodies or sorted to protein storage vacuoles (Pedrazzini et al., 2016; Ashnest and Gendall, 2018). Protein bodies are storage ERDVs that are widely present in cereal plants but have been best characterized in the endosperm of maize, which accumulates seed storage proteins called zeins (Holding, 2014; Larkins, 2019). Zeins belong to the class of seed storage proteins called prolamins, which also accumulate in endosperm of other cereals such as rice. In rice endosperm, prolamins are also stored in ER‐derived protein bodies (also referred to as protein body I) (Tanaka et al., 1980). By contrast, another class of rice storage proteins known as glutelins are synthesized on the ER but transported to protein storage vacuoles (also known as protein body II) through a Golgi‐dependent pathway (Tanaka et al., 1980; Krishnan et al., 1986).

Another type of storage ERDV is precursor‐accumulating (PAC) vesicles found in maturing seeds of pumpkin (Hara‐Nishimura et al., 1985, 1993). PAC vesicles accumulate precursors of storage proteins 2S albumin and 11S globulin to be transported to protein storage vacuoles (Hara‐Nishimura et al., 1985, 1993). After deposition in the vacuoles, these storage protein precursors are processed to the mature forms by vacuole‐specific enzymes. These storage protein precursors likely form aggregates upon synthesis on the ER and develop into PAC vesicles for direct trafficking into the protein storage vacuole in a Golgi‐independent manner (Hara‐Nishimura et al., 1998). The precursor of a novel membrane protein, MP73, is also transported to and processed in protein storage vacuoles through PAC vesicles (Mitsuhashi et al., 2001). The vacuolar sorting receptor PV72 was found on the membrane of the PAC vesicles and binds to the C‐terminal vacuolar targeting signal of 2S albumin precursor in pumpkin seeds and, therefore, may mediate the transport of the storage protein to the storage vacuoles (Shimada et al., 2002; Watanabe et al., 2002). Interestingly, despite the Golgi‐independent nature of the PAC vesicle trafficking, a green fluorescent protein (GFP) fusion protein with the transmembrane domain and the cytosolic tail of PV72 was localized in the Golgi apparatus (Shimada et al., 2002). It has been proposed that some storage protein precursors may leave the ER for the Golgi apparatus but are recruited back to the PAC vesicles through the action of the vacuolar sorting receptor PV72 (Shimada et al., 2002) (Figure 1). However, there is also evidence for recycling of vacuolar sorting receptors to the ER for cargo binding (Robinson and Neuhaus, 2016) and, therefore, the PV72 on the PAC vesicles could also originate from the ER.

### Hydrolytic enzyme ERDVs: PPVs

ER‐derived hydrolytic enzyme vesicles accumulate hydrolases such as proteases and glycosidases. One type of hydrolytic enzyme ERDVs are known as precursor protease vesicles (PPVs) from mung bean (*Vigna radiata*) or ricinosomes from caster bean (*Ricinus communis*) that accumulate *de novo* synthesized precursors of papain‐type Cys proteases for the proteolysis of proteins in the storage tissues (e.g., cotyledons) of growing seedlings (Mollenhauer and Totten, 1970; Chrispeels et al., 1976; Baumgartner et al., 1978; Schmid et al., 1998). In seedlings of black gram (*Vigna mungo*), the degradation of cotyledon storage proteins in the protein storage vacuole is dependent on the biosynthesis of a papain‐type Cys protease called sulfhydryl‐endopeptidase (SH‐EP) (Toyooka et al., 2000). SH‐EP pro‐protease ends with a KDEL‐ER‐retention motif at its C‐terminus and upon synthesis from the ER, can accumulate in a type of ERDVs known as KDEL‐tailed Cys protease‐accumulating vesicles (KVs) (Toyooka et al., 2000), which are likely to be identical to PPVs and ricinosomes from other legume species. The KDEL‐tailed Cys proteases accumulated in ER‐derived PPVs are then processed into mature and active 33‐kD protease through several intermediates including the removal of the KDEL tail during or after its transport to the protein storage vacuoles (Toyooka et al., 2000). Immunoelectron microscopy of the cotyledon cells of germinating black gram seeds using anti‐SH‐EP antibodies detected accumulation of the Cys protease at the ER and KVs but not in the Golgi complex (Toyooka et al., 2000). By contrast, immunoelectron microscopy using antibodies to the complex glycans detected the Asn‐linked Golgi glycosylation products in the Golgi complex and protein storage vacuole, but not in the KVs (Toyooka et al., 2000). These results indicate that the SH‐EP Cys protease is transported to the protein storage vacuoles by ER‐derived KVs in a Golgi‐independent manner.

Even though PPVs, ricinosomes and KVs, which all accumulate KDEL‐tailed Cys proteases, were initially identified during seed germination of legume plants and may contribute to storage protein degradation and mobilization through direct proteolytic degradation, there is strong evidence that these proteases play important roles in regulation of developmentally regulated PCD. After oil and protein reserves in the storage tissues have been mobilized during germination of castor bean seeds, the cells of the endosperm undergo PCD, which is associated with nuclear DNA fragmentation. The initiation of PCD in the endosperm is associated with release of mature and active Cys proteases into the cytoplasm by the ricinosomes (Schmid et al., 1999). Acidification of isolated ricinosomes causes the cleavage of the N‐terminal propeptide and the C‐terminal KDEL motif of the castor bean Cys endopeptidases, leading to their activation (Schmid et al., 2001). It has been proposed that inactive Cys protease precursors accumulate in ricinosomes in the endosperm during germination and are activated by acidification of the cytoplasm from the disruption of the vacuole to promote PCD in the final stages of endosperm disintegration (Gietl and Schmid, 2001; Schmid et al., 2001; Greenwood et al., 2005; Lopez‐Fernandez and Maldonado, 2013). The KDEL Cys proteases from caster bean accept a wide variety of amino acids at the active site and can digest the hydroxyproline (Hyp)‐rich proteins (extensins), the basic scaffold of the plant cell wall (Helm et al., 2008).

Genes encoding KDEL‐tailed Cys proteases are present in all plants (Hierl et al., 2012). In *Arabidopsis*, there are three genes for KDEL‐tailed cysteine endopeptidases (AtCEP1, 2 and 3). Molecular and genetic analysis has revealed critical roles of *Arabidopsis* KDEL‐tailed Cys proteases in regulation of PCD in senescing tissues. These Cys protease genes are expressed not only during seed germination, but also during flower and root development, particularly during the final stages of PCD in collapsing tissues (Helm et al., 2008). *AtCEP1* is also expressed in the tapetum from Stages 5 to 11 of anther development (Zhang et al., 2014). AtCEP1 protein is detected first as a proenzyme in PPVs and processed into the active mature enzyme after transport to the vacuole before its rupture (Zhang et al., 2014). *Arabidopsis atcep1* mutants display aborted tapetal PCD and reduced pollen fertility associated with abnormal pollen exine (Zhang et al., 2014). Transcriptomic analysis showed that mutation of *AtCEP1* affected expression of genes important for tapetal cell wall organization, tapetal secretory structure formation, and pollen development (Zhang et al., 2014). By contrast, *AtCEP1* overexpression leads to premature tapetal PCD and pollen infertility (Zhang et al., 2014). These results reveal that AtCEP1 plays a critical role in tapetal PCD for pollen grain development. A similar role of ricinosomes and Cys proteases in PCD during anther dehiscence has also been reported in tomato (Senatore et al., 2009). *Arabidopsis* AtCEP1 also regulates PCD of both tracheary elements and fiber cells during xylem development. *AtCEP1* expression levels is elevated in inflorescence stems during stem maturation and the Cys protease can be detected in the cell wall of xylem cells (Han et al., 2019). Mutations of *AtCEP1* delay stem growth and reduce xylem cell number, which is associated with delayed organelle degradation during PCD, and increased thickness of secondary walls in tracheary elements and fiber cells (Han et al., 2019). Mutation of *AtCEP1* increases expression of genes involved in the biosynthesis of secondary wall components, including cellulose, hemicellulose, and lignin (Han et al., 2019). The mutation of *AtCEP1* also elevates the expression of wood‐associated transcriptional factors in the maturation stage of the inflorescence stem (Han et al., 2019). Thus, AtCEP1 is a positive regulator of the mobilization of cellular content during PCD but a negative regulator of the secondary wall thickening during xylem development.

PCD plays an important role in plant–pathogen interaction, particularly during the rapid hypersensitive response at the site of infection, which limits the spread of biotrophic pathogens (Li et al., 2020). *AtCEP1* expression is responsive to biotic stresses in leaves (Howing et al., 2014, 2017). Mutations of *AtCEP1* enhance susceptibility to powdery mildew caused by the biotrophic ascomycete *Erysiphe cruciferarum*. The *atcep1* mutants also display deregulated expression of stress response genes during their interaction with *E. cruciferarum* (Howing et al., 2014, 2017). Based on the analysis of spatiotemporal *AtCEP1*‐reporter expression during fungal infection and the microscopic inspection of the interaction phenotype, AtCEP1 functions in restriction of powdery mildew likely through controlling latest ages of compatible interaction including late epidermal cell death, implicating AtCEP1 as a regulator of pathogen‐induced PCD during plant interaction with biotrophic pathogens (Howing et al., 2014, 2017).

Proteases are key regulators and executors of PCD in animals (Moffitt et al., 2010). The most prominent proteases in animal PCD are Cys‐dependent aspartate‐specific proteases known as caspases. The molecular hallmark of PCD, or apoptosis is the activation of caspases. Caspases are synthesized as relatively inactive zymogens and undergo activation during apoptosis (Poreba et al., 2013). There are two families of caspases that differ in their order of activation: the initiator caspases and the effector caspases (Poreba et al., 2013). The initiator caspases undergo a complex process of autocatalytic processing and activation in response to upstream apoptotic stimuli. An activated initiator caspase can specifically cleave and activate an effector caspase zymogen (Poreba et al., 2013). There are no caspase homologs in plants (Uren et al., 2000) but other families of proteases including KDEL‐tailed Cys proteases have important roles in the regulation and progression of developmentally regulated or stress‐induced PCD processes in plants (Buono et al., 2019). Apparently, like caspases in animals, KDEL‐tailed Cys proteases in plants also involve an elaborate scheme of activation to promote PCD. In both the endosperm of germinating seeds, the tapetum in the anther and xylem cells, ER‐synthesized Cys proteases are stored in PPVs as an inactive proenzyme but are activated into mature enzymes upon initiation of PCD either after transport to the vacuole before its rupture or by acidification of the cytoplasm resulting from the disruption of the vacuole. Therefore, specialized ERDVs serve as reserve vesicles for inactive Cys protease proenzymes that can be activated and deployed upon PCD initiation in the endosperm, tapetum and xylem cells (Figure 1).

### Hydrolytic enzyme ERDVs: ER bodies

ER bodies are produced only by plants in the Brassicales order, including *Arabidopsis* (Nakano et al., 2014). Unlike other ERDVs, ER bodies are rod‐shaped, approximately 1 μm in diameter and 10 μm in length and can be observed in transgenic *Arabidopsis* plants expressing ER‐targeted GFP (Hawes et al., 2001; Hayashi et al., 2001). Analysis using electron microscopy showed that the ER bodies contain a single membrane covered by ribosomes and are connected with ER tubules and cisternae, indicating that the ER bodies are continuous to the whole ER network (Hayashi et al., 2001). ER bodies are generally classified into two types: (i) constitutive ER bodies in the epidermal cells of the cotyledons, hypocotyls and roots of *Arabidopsis* seedlings and (ii) wound/jasmonic acid (JA)‐inducible ER bodies in the rosette leaves. More recently, a third type of ER body called leaf ER bodies has been reported to be constitutively present in specific cells of rosette leaves (marginal cells, epidermal cells covering the midrib and giant pavement cells) (Nakazaki et al., 2019). The major protein component of the constitutive ER bodies in *Arabidopsis* is PYK10/BGLU23, a β‐glucosidase with a KDEL‐ER‐retention signal at its C terminus (Matsushima et al., 2003b). Two integral membrane proteins with a metal ion transporter activity, MEMBRANE OF ER BODY1 (MEB1) and MEB2, have also been identified to accumulate specifically at the membranes of constitutive ER bodies in *Arabidopsis* (Yamada et al., 2013). Wound‐inducible ER bodies, on the other hand, accumulate primarily BGLU18 (Ogasawara et al., 2009), another member of the KDEL‐tailed β‐glucosidase family, whereas leaf ER bodies contain both PYK10/BGLU23 and BGLU18 (Nakazaki et al., 2019).

*Arabidopsis* contains eight KDEL‐tailed BGLU proteins (BGLU18 to 25). Biochemical analysis indicates that the abundant BGLU proteins in the ER bodies has a myrosinase activity that hydrolyzes glucosinolates, thereby generating chemically reactive products toxic to pathogens and herbivores (Nakano et al., 2017). Like ER bodies, glucosinolates are produced only by plants in the Brassicales order and are critical components of a chemical defense system called the mustard oil bomb in these plants (Matile, 1980; Luthy and Matile, 1984). In mature leaves of *Arabidopsis*, the mustard bomb acts through a dual‐cell type mechanism in which glucosinolates and myrosinases accumulate in two different types of cells but can get access to each other upon tissue damage, leading to hydrolysis of glucosinolates and production of toxic isothiocyanates (Shirakawa and Hara‐Nishimura, 2018). In the seedlings, apparently, the mustard bomb operates through a single‐cell mechanism in which a different family of myrosinases and glucosinolates are stored in ER bodies and vacuole, respectively, in the same cells and gain access to each other upon tissue damage to produce toxic products (Yamada et al., 2020) (Figure 1). Significantly, genes associated with the ER body, glucosinolate biosynthesis and metabolism display a striking co‐expression pattern, suggesting strong coordination among these processes (Nakano et al., 2017). The role of ER bodies in plant chemical defense has been supported by the finding that *Arabidopsis* unable to form ER bodies is hypersusceptible to herbivores such as woodlice and the chewing insect *Spodoptera exigua* (Yamada et al., 2020; Rufian et al., 2021). The ER body‐deficient mutants also leads to overgrowth of the beneficial fungus *Piriformospora indica* without beneficial effects on the plants (Sherameti et al., 2008). This suggests that ER body formation plays a role in plant defense that enables controlled fungal colonization to establish a mutualistic interaction between the symbiotic partners (Sherameti et al., 2008). Interestingly, ER bodies are induced by the bacterial pathogen *Pseudomonas syringae* in a manner dependent on the bacterial toxin coronatine but play a negative role in immunity against the bacterial pathogen (Rufian et al., 2021). Thus, the bacterial pathogen exploits the ER bodies as a counter‐defense mechanism to promote virulence. The ER body may also play a role in plant responses to other stresses, including drought and metal ion toxicity (Yamada et al., 2013; Kumar et al., 2015).

Genetic analysis has identified two genes, *NAI1* and *NAI2*, with an important role in the ER body formation in *Arabidopsis* (Matsushima et al., 2004; Yamada et al., 2008). *NAI1* encodes a basic helix‐loop‐helix (bHLH)‐type transcription factor and functions as a master regulator of the ER body formation by regulating the expression of genes associated with ER bodies including PYK10/BGLU23, *NAI2*, MEB1 and MEB2 (Matsushima et al., 2004). *NAI2* encodes an ER body component that determines the constitutive ER body formation in *Arabidopsis* (Yamada et al., 2008). In the *nai2* mutants, PYK10/BGLU23, MEB1 and MEB2 are diffused throughout the ER and the levels of PYK10 are reduced, indicating that NAI2 promotes accumulation of PYK10 by mediating the formation of the ER bodies (Yamada et al., 2008). NAI2 forms complexes with MEB1 and MEB2 and, therefore, may be responsible for the recruitment and organization of these ER body cargo proteins (Yamada et al., 2013). In *Arabidopsis*, NAI2 has a close homolog, TONSOKU (TSK)‐ASSOCIATED PROTEIN1 (TSA1), which plays a critical role in wound/JA‐induced ER body formation (Geem et al., 2019). Like ER bodies and glucosinolates, NAI2 homologs are found only in plants in the Brassicaceae order, suggesting that NAI2 and its homologs have evolved specifically for the formation of the ERDVs (Yamada et al., 2008).

## EVOLUTIONARY ORIGIN OF SPECILIZED ERDVS

### Evolutionary mechanisms for the protein body formation

Among the specialized ERDVs that have been analyzed, some including protein bodies and ER bodies are associated with or unique to certain orders or families of plants. Protein bodies are a major storage organelle of seed proteins in the Poaceae family (commonly known as grasses), which originated relatively recently (Gaut, 2002), and have been extensively analyzed in cereal plants, particularly in maize and rice. ER bodies are produced only by plants in the Brassicales order and have been almost exclusively analyzed in *Arabidopsis*. The association of these specialized ERDVs with specific groups of plants raises important questions about their evolutionary origin. Analysis of the key determinants for the formation of protein bodies in cereal plants, ER bodies and related ERDVs in *Arabidopsis* has provided important insights into the evolutionary events that led to these remarkable subcellular structures and functions in plants.

Protein bodies are ER‐derived compartments that accumulate prolamin storage proteins in the endosperm cells of cereal seeds. Prolamin storage proteins rapidly form very large and insoluble polymers in the ER upon synthesis due to inter‐chain disulfide bonds and hydrophobic interactions (Pedrazzini et al., 2016). Insoluble protein aggregates are likely to be secretion incompetent as they are unable to pass through the elaborate ER protein quality control system and their massive accumulation in the ER would cause severe ER stress (Granell et al., 2008; Ito et al., 2012). Therefore, rapid segregation and accumulation of prolamins in physically separated ER‐derived protein bodies are likely an adaptive mechanism to reduce the toxic effects of a massive amount of insoluble protein polymers to protect cell survival without compromising accumulation of the storage proteins. Consistent with this hypothesis, protein bodies can be induced by prolamin proteins such as 27 KD γ‐zein and its fusions with other proteins not only in non‐cereal plants but also in fungal, mammalian, insect and yeast cells (Torrent et al., 2009; Reifenrath et al., 2020). There is also evidence that protein bodies in plants are similar to the ER‐derived Russell bodies in mammalian cells that result from the accumulation of aggregated proteins from misfolded or abundant proteins in the ER (Arcalis et al., 2019), even though the mechanisms for the formation of ordered heteropolymeric storage proteins in protein bodies are very different from those of misfolded protein aggregates. Therefore, the molecular machineries for formation of protein bodies are ubiquitously present in eukaryotic cells. A key factor that led to the formation of protein bodies in the endosperm cells of cereal seeds is the structural changes and development of special polymeric features of prolamin storage proteins during the evolution of grasses (Shewry and Halford, 2002).

**Figure 2:**
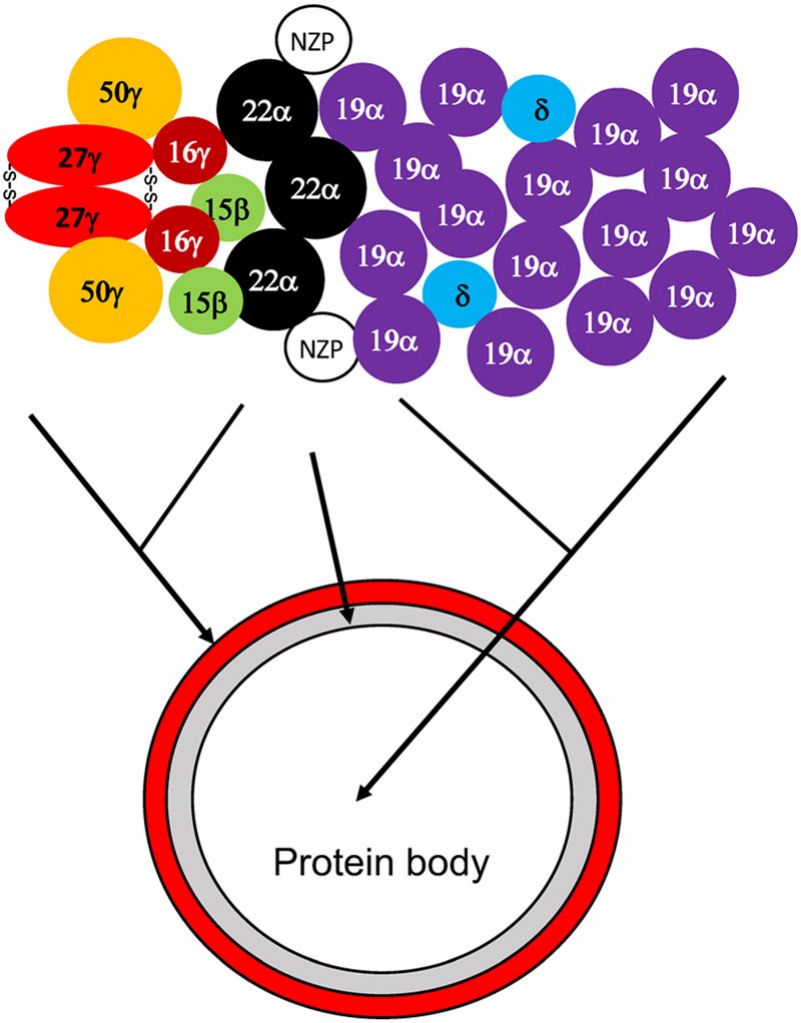
A schematic diagram of zein protein interaction and organization in the protein bodies The 27 kD γ‐zeins polymerize through inter‐chain disulfide bonds and protein–protein interaction and play a fundamental role in the protein body formation. Other γ‐zeins are sequestered into the protein bodies through interaction with the 27 kD γ‐zeins and together form the peripheral shell of the protein bodies. The 16 kD γ‐zeins interact with both the 27 kD γ‐zeins and 15 kD β‐zeins and both can interact with the 22 kD α‐zeins, which form the intermediary layer of the protein bodies. The 19 kD α‐zeins are the most abundant class of zeins and together with δ‐zeins form the interior of the protein bodies. Non‐zein proteins (NZP) are also sequestered in the protein bodies through interaction with α‐zeins.

Prolamins likely evolved from a soluble cereal α‐globulin (Xu and Messing, 2008, 2009), which, unlike prolamins, is transported through the usual ER‐Golgi secretory pathway and stored in protein storage vacuoles. Prolamins, α‐globulin and other proteins in the prolamin superfamily share a common domain derived from the eight‐Cys motif (8CM) with eight specifically ordered Cys residues in three conserved regions termed A, B and C that generate four intra‐chain disulfide bridges (Kreis et al., 1985). Protein bodies in maize accumulate four types of zeins, α‐ (19 and 22 kD), β‐ (15 kD), γ‐ (16, 17 and 50 kD) and δ‐ (10 and 18 kD) zeins. These zein proteins differ in their structures and, consequently, in the way they accumulate in protein bodies. The 27 kD γ‐zein plays a fundamental role in protein body formation and can itself form protein bodies when expressed in transgenic plants (Lending and Larkins, 1989). The feature of extensive polymerization of 27 kD γ‐zein is largely attributed to inter‐chain disulfide bridges and hydrophobic interaction. The Cys residues in the 8CM motif of maize 27 kD γ‐zein remain largely intact during its evolution but the maize storage protein contains an N‐terminal domain composed of eight repeats of a VHLPPP necessary for ER retention and seven additional Cys residues that can generate inter‐chain disulfide bonds (Pedrazzini et al., 2016) (Figure 2). Both the VHLPPP repeats and the additional Cys residues of the 27‐kD γ‐zein are required for protein body formation (Llop‐Tous et al., 2010; Mainieri et al., 2014). The 16‐kD γ‐zein, which probably originates from of the 27‐kD γ‐zein gene, lacks some of the Pro‐rich repeats and the Cys residues involved in inter‐chain bonds. As a result, the 16‐kD γ‐zein is partially soluble and unable to induce protein bodies (Mainieri et al., 2018). The 16 kD γ‐, α‐, β‐ and δ‐zeins, therefore, rely on the 27 kD γ‐zein proteins for sequestering and accumulation in protein bodies through extensive protein–protein interaction. The 16‐kD γ‐zein can interact with all classes of zeins including the 27 kD γ‐zein and abundant α‐zeins localized in the interior part of protein bodies (Kim et al., 2002; Holding, 2014; Mainieri et al., 2018) (Figure 2). The 15‐kD β‐zein is related to γ‐zeins and can also interact with δ‐ and highly abundant α‐zeins, in addition to its interaction with the 16‐kD γ‐zein (Kim et al., 2002) (Figure 2). Therefore, while the 27 kD γ‐zein is key to protein body formation, the 16‐kD γ‐ and 15‐kD β‐zeins play a special role in the recruitment and sequestering of δ‐ and highly abundant α‐zeins for the assembly of natural heteropolymeric protein bodies (Figure 2).

In addition to zeins, protein bodies in maize accumulate other proteins including FLOURY1, a novel ER protein involved in zein protein body formation (Holding et al., 2007), and OPAQUE10, a cereal‐specific protein required for distribution of zeins in endosperm protein bodies (Yao et al., 2016). Proteomic profiling of artificial protein bodies induced by a γ‐zein fusion protein in *Nicotiana benthamiana* leaf cells and natural protein bodies isolated from maize endosperm identified 195 and 2 283 proteins, respectively, with diverse biological functions and various subcellular localizations, including the nucleus, cytosol, chloroplasts, mitochondria, and ER (Joseph et al., 2012; Wang et al., 2016). Very recently, it has been reported that the mitochondrial 50S ribosomal protein L10 (mRPL10) is localized not only to mitochondria but also to protein bodies as a non‐zein protein (Feng et al., 2021). Importantly, the accumulation of the maize non‐zein protein in the protein bodies is depended on its interaction with α‐zeins (Feng et al., 2021) (Figure 2). Another non‐zein protein with plastidial localization also accumulated in induced protein bodies through interaction with α‐zeins (Feng et al., 2021). Therefore, non‐zein proteins are recruited to protein bodies through interaction with zein proteins (Figure 2). Some of these non‐zein proteins contain no N‐terminal signal peptides required for their translocation across the ER membrane to accumulate in ER‐derived protein bodies, raising the possibility of unconventional trafficking of specialized ERDV cargo proteins.

**Figure 3:**
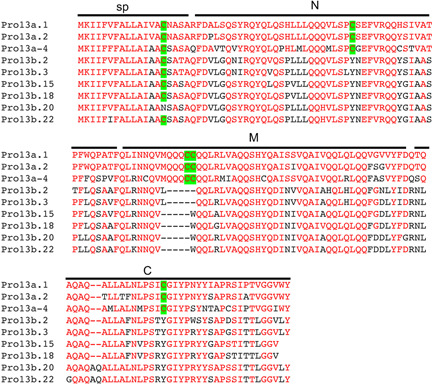
Protein sequence alignment among rice 13a and 13b prolamins The signal peptide (sp) and the N‐terminal (N), middle (M) and C‐terminal (C) regions corresponding to the A, B and C regions, respectively of the C8M motif in the prolamin superfamily proteins are indicated. The amino acid residues in the proteins identical to those in rice 13a.1 prolamin (Pro13a.1) are in red. The Cys residues in these proteins are also highlighted.

Prolamins from other cereal plants acquired the features of extensive polymerization during evolution also by developing inter‐chain disulfide bridges and hydrophobic interactions but through structural changes different from that of maize 27‐kD γ‐zein. For wheat high molecular weight prolamins, a very large Pro‐ and Gln‐rich repetitive domain was inserted into the hypervariable loop between B and C regions of the 8CM motif, while rice 13a prolamins underwent sequence deletion within the regions (Kawagoe et al., 2005; Onda et al., 2011). It has been proposed that the altered length and additional sequence changes resulted in increased formation of inter‐chain disulfide bonds by the Cys residues in the 8CM motif at the expense of intra‐chain disulfide bonds, leading to increased prolamin polymerization (Kawagoe et al., 2005; Onda et al., 2011). Rice 13a prolamin contains four Cys residues and its GFP fusion can form protein bodies in yeast cells (Masumura et al., 2015) (Figure 3). Deletion analysis with GFP fusions indicated that the middle and C‐terminal region of rice 13a prolamin, which corresponds to the B and C regions of the 8CM motif, form structures similar to protein bodies in yeast. By contrast, the N‐terminal region of rice 13a prolamin, which corresponds to the A region of the 8CM motif, did not form protein bodies and its deletion did not affect the protein body formation. Therefore, the Cys residues in the B and C domains are important for protein body formation, mostly likely through inter‐chain disulfide bridges that promote polymerization. Like protein bodies in maize, rice protein bodies contain multiple types of prolamins including Cys‐rich 10‐kD, 13a, 16 and Cys‐poor 13b prolamins (Saito et al., 2012; Sasou et al., 2018). Both Cys‐rich 10 and 13a prolamins are concentrated at the electron‐dense center core and middle regions of protein bodies and play a crucial role in the formation of the initial rice protein body core (Nagamine et al., 2011; Masumura et al., 2015). Rice 13b prolamins, on the other hand, contain the conserved Cys residue in the N‐terminal region corresponding to the A region of the 8CM motif but lack the Cys residues in the B and C regions (Figure 3). Rice 13b prolamins, which are distributed mainly to the electron‐lucent peripheral region of protein bodies, join the storage protein structures most likely through protein–protein interactions (Onda and Kawagoe, 2011). Thus, formation of protein bodies in different cereal seeds all rely on specific prolamins such as maize 27‐kD γ‐zein capable of forming inter‐chain disulfide bonds to initiate the core of protein bodies, and then recruit other prolamins or even non‐prolamin proteins that are unable to form protein bodies themselves through direct and indirect protein–protein interaction.

### Evolutionary origin of ER bodies

Unlike protein bodies from cereal plants, formation of the ER bodies in Brassicaceae plants require specific factors such as NAI2 that are only present in these plants (Yamada et al., 2008). An important evolutionary question about the ER bodies is whether they originated in Brassicaceae plants or evolved from pre‐existing ER structures. From the analysis of three closely related NAI2‐interacting proteins (NAIP1, 2 and 3), we have provided important insights into the evolutionary origin of the ER bodies (Wang et al., 2019a). The NAIP proteins were initially identified for their interaction with a conserved ER‐resident protein, UBAC2, with roles in protein quality control and selective autophagy of the ER (Zhou et al., 2018; Wang et al., 2019a, 2019b; Li et al., 2021). The NAIPs are most conserved at their C‐terminal regions homologous to the protein‐binding harmonin homology domain (HHD). The three proteins are also similar at the N‐terminal coiled‐coil (CC) domains. The middle parts of NAIPs are highly divergent but all contain multiple TP or SP phosphorylation motifs by so‐called proline‐directed protein kinases including cyclin‐dependent protein kinases and mitogen‐activated protein kinases (Lee et al., 2005). Thus, the NAIP proteins are rich in protein‐interacting motifs and are potentially regulated by protein phosphorylation. Homologs of NAIP genes are not present in the archaea, eubacteria, fungi or animals but found in the kingdom of Protista, most belonging to the phylum of Apicomplexa in the large clade of parasitic alveolates (Wang et al., 2019a). Importantly, NAIP homologs are found in all plants including the unicellular green alga *Chlamydomonas reinhardtii*, the moss *Physcomitrella patens*, the fern *Selaginella moellendorffii* and both angiosperms and gymnosperms. Thus, NAIP proteins have originated in early eukaryotes and are present in all branches of land plants usually as a small family with three to four paralogs (Wang et al., 2019a).

**Figure 4:**
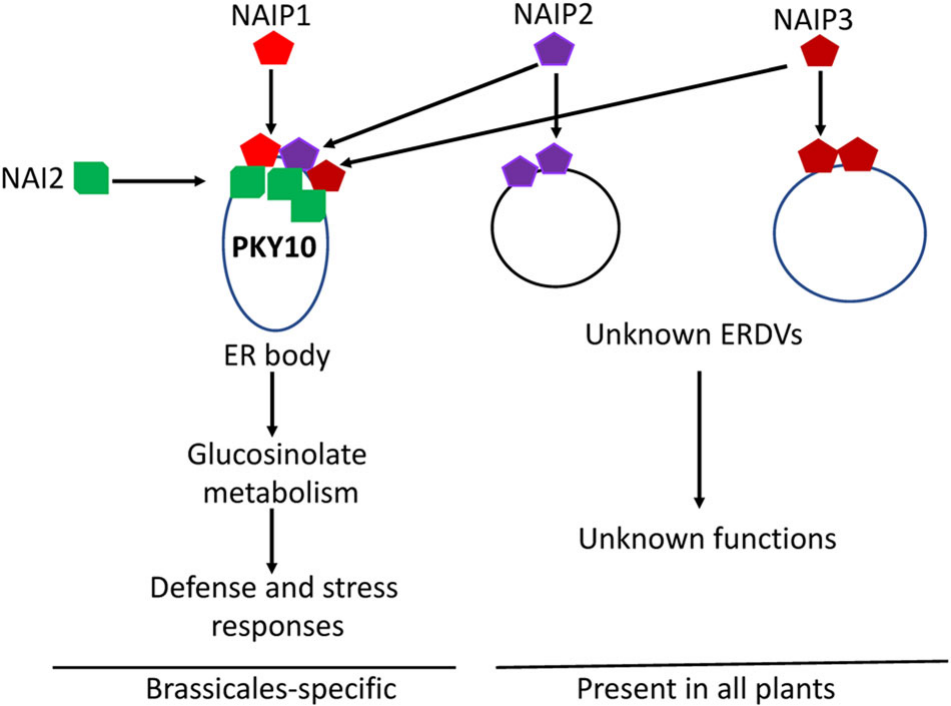
Roles of NAI2‐interacting NAIP proteins in the formation of endoplasmic reticulum (ER) bodies and related ER‐derived vesicles (ERDVs) NAIP1 is specifically associated with ER bodies through interaction with NAI2. ER bodies accumulate a family of β‐glucosidases such as PYK10 with a myrosinase activity, which can hydrolyze glucosinolates in defense and stress responses. NAIP2 and NAIP3 are associated with both ER bodies but also other unknown ERDVs the formation of which is not dependent on NAI2. These NAIP2‐ and NAIP3‐containing ERDVs are present in all plants but their cargo proteins and biological functions are unclear.

Genetic analysis indicates a critical and redundant role of the NAIPs in the formation of the ER bodies (Wang et al., 2019a). Constitutive ER body formation is normal in the *naip* single and *naip1/naip2* double mutants but is almost completely abolished in the *naip1/naip2/naip3* triple mutant, as in the *nai2* mutant (Wang et al., 2019a). Studies using the GFP fusion constructs further revealed that NAIP1 formed punctate structures in a tissue‐specific pattern identical to those of known ER body markers and the formation of the NAIP1‐GFP punctate structures is NAI2‐dependent, indicating that NAIP1 is specifically associated with the ER bodies(Wang et al., 2019a). On the other hand, NAIP2‐ and NAIP3‐GFP fusion proteins formed punctate structures not only in the cotyledons, hypocotyls and roots where constitutive ER bodies are formed but also in the rosette leaves where constitutive ER bodies are not present (Wang et al., 2019a). In addition, formation of punctate structures by NAIP2‐ and NAIP3‐GFP fusion proteins is not NAI2‐dependent (Wang et al., 2019a). Thus, unlike NAIP1, NAIP2 and NAIP3 are associated not only with the ER bodies but also with other vesicular structures the formation of which is ubiquitous and NAI2 independent. Based on these findings, we have proposed that the NAI2/TSA1‐containing ER bodies in the Brassicales may have evolved from NAIP‐containing ER‐derived structures widely present not only in plants but also in protists (Wang et al., 2019a) (Figure 4). In *Arabidopsis*, while NAIP1 has evolved to function specifically for ER body formation, NAIP2 and NAIP3 are less specialized and can function as components of not only the ER bodies but also other ER‐derived structures that can be formed in a wider range of plant tissues (Wang et al., 2019a) (Figure 4).

**Figure 5:**
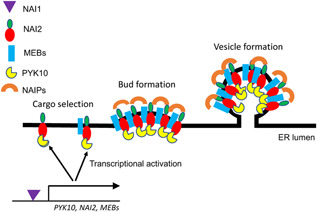
A model for the roles of NAI1, NAI2 and NAIP proteins in the biogenesis of endoplasmic reticulum (ER) bodies NAI1 is a transcription factor that regulates the expression of genes encoding PYK10, NAI2 and MEMBRANE OF ER BODY (MEBs). NAI2 plays a role in the recruitment of cargo proteins such as PRK10 and membrane protein MEBs through protein–protein interactions. NAI2 also recruits NAIP proteins to the surface of the ER membrane through its C‐terminal domain, which interacts with the C‐terminal harmonin homology domains (HHDs) of NAIPs. At the membrane surface, the NAIP proteins could further assemble through self‐interaction and interaction with other proteins via their N‐terminal CC domain to coordinate or promote cargo recruitment, deformation and budding of the membrane to drive the formation of the ER‐derived vesicles (ERDVs).

NAI2, its paralog TSA1 and their interacting protein NAIPs are, to our knowledge, the only known proteins to be both associated with specialized ERDVs and required for their formation. These proteins, therefore, could play a direct role in cargo recognition, vesicle budding and transport of their associated ERDVs. Formation of well‐characterized clathrin vesicles, which also carry specific proteins, involve recruitment of the G‐protein ARF, adaptor proteins and clathrin to defined sites on the membrane, where adaptor protein‐specified assembly of clathrin, formation of clathrin‐coated pits and cargo recruitment takes place, followed by membrane deformation, budding and detachment of the nascent clathrin‐coated vesicles (Paraan et al., 2020). Both NAI2 and TSA1 contain a signal peptide at their N terminus to enter the ER lumen and are localized in ER bodies (Stefanik et al., 2020). NAI2 and TSA1 share a similar domain organization consisting of a N‐terminal 10 Glu‐Phe‐Glu (EFE) repeats with Ca^2+^‐binding activity, a putative transmembrane domain, and a C‐terminal protein‐interacting domain (Suzuki et al., 2005; Wang et al., 2019a). On the other hand, the NAIP proteins contain no predicted transmembrane domain or signal peptide and are likely to be localized on the cytosolic side of ER bodies (Wang et al., 2019a). The NAIP proteins can interact with themselves or with each other through their C‐terminal CC domains and interact with the C‐terminal domains of NAI2 and TSA1 through their C‐terminal HHD domains (Wang et al., 2019a). The ER lumen‐localized EFE repeats at the N‐terminal region of NAI2 and TSA1 can mediate formation of multimeric complexes but could also be involved in cargo selection, possibly in a Ca^2+^‐sensitive manner, in the ER lumen during the early stages of ER body biogenesis (Suzuki et al., 2005) (Figure 5). The demonstration that NAI2 forms protein complexes with ER membrane proteins MEB1 and MEB2 is consistent with its role in cargo recruitment (Yamada et al., 2013). In addition, both NAI2 and TSA1 may function as adaptors through their C‐terminal domains to recruit NAIPs to the membrane surface through interaction with the C‐terminal HHD domains of NAIPs (Figure 5). At the membrane surface, the NAIP proteins could further assemble through self‐interaction and interaction with other proteins via their N‐terminal CC domain to coordinate or promote cargo recruitment, deformation and budding of the membrane to drive the formation of the ERDVs (Figure 5).

## BIOTECHNOLOGICAL EXPLOITATION OF PLANT ERDVs

Production of recombinant proteins including enzymes, vaccines, antibodies, and other therapeutic proteins in plants is an area of great potential because of important benefits in safety, cost and efficiency (Kopertekh and Schiemann, 2019; Nosaki and Miura, 2021). High production yield and efficient purification are two main challenges to overcome for any platform to become efficient for production of recombinant proteins. Besides improving expression of transgenes for foreign proteins, there have been efforts to target recombinant proteins to different subcellular compartments, such as the ER, extracellular space and chloroplasts to promote their accumulation in plants (Habibi et al., 2017). Protein bodies accumulate massive levels of storage proteins and, therefore, have also been studied as storage organelles for high levels of recombinant proteins in plant cells. Even though protein bodies are normally produced in storage tissues, they can be induced artificially in other tissues including leaves by overexpression of protein body‐inducing proteins or their fusion with a recombinant protein. The ease to induce protein bodies in plant leaves makes it possible to rapidly produce recombinant proteins through transient gene expression in *N. benthamiana* (Kopertekh and Schiemann, 2019; Nosaki and Miura, 2021).

Three types of protein fusion tags have been widely used to target recombinant proteins into protein bodies with positive effect on their accumulation in plant cells: Zera, elastin‐like polypeptide (ELP) and hydrophobins (HFBs). Zera is a peptide of 112 residues composed of the signal peptide and N‐terminal proline‐rich domain of γ‐zein with six cysteine residues capable for forming inter‐chain disulfide bonds, which promote oligomerization of Zera molecules, formation of protein bodies and accumulation of fused recombinant proteins (Kogan et al., 2001; Mainieri et al., 2004; de Virgilio et al., 2008; Llop‐Tous et al., 2010). Purification of Zera‐fused proteins is facilitated by isolation of induced protein bodies using density‐based centrifugation. Zera fusions have been used for production and purification of recombinant human growth hormone, epidermal growth factor and Streptomyces derived xylanases (Llop‐Tous et al., 2010, 2011). ELPs are synthetic biopolymers with a general structure of VPGXG repeats (X can be any non‐proline amino acid) originally identified in the mammalian protein elastin (Urry,1988a, 1988b). ELPs share structural characteristics with intrinsically disordered proteins and undergo a reversible phase transition from soluble protein to insoluble aggregates above specific transition temperatures (Roberts et al., 2015). This property of ELPs can be used for rapid purification using a procedure known as inverse transition cycling. An ELP peptide of 30–40 VPGXG repeats increases accumulation of recombinant proteins such as spider silk proteins, murine interleukin‐4 (Patel et al., 2007), human interleukin‐10 (Kaldis et al., 2013), anti‐HIV antibody 2F5 (Floss et al., 2008), and neutralizing antibodies against H5N1 virus (Phan et al., 2013). In *N. benthamiana* leaves, ELP fusion to GFP substantially increased the amount of GFP accumulation (up to 40% of total soluble proteins) and this increase was associated with induced formation of GFP‐containing protein bodies (Saberianfar et al., 2015). HFBs are a family of small, secretory proteins produced by filamentous fungi (Linder et al., 2005). HFBs are globular proteins stabilized by four disulfide bonds with a hydrophobic patch on the surface, giving rise to their hydrophobic and extraordinarily surface‐active properties. These properties of HFBs can be transferred to their fusion proteins and used for purification using aqueous two‐phase separation (Linder et al., 2004). When used as a fusion tag, HFB1 from *Trichoderma reesei* can increase the accumulation of glucose oxidase, which is difficult to express with other expression systems (Bankar et al., 2009). HFBI as a fusion tag also improved accumulation of GFP up to 51% of the total soluble protein and increased the yield of other target proteins in plants. When transiently expressed in *N. benthamiana* leaves, HFBI‐GFP fusion induced the formation of clustered plant bodies (Joensuu et al., 2010).

Protein bodies can also be induced in non‐plant eukaryotes. Various Zera fusions with fluorescent and therapeutic proteins including calcitonin, epidermal growth factor and human growth hormone, induce protein body‐like organelles not only in tobacco leaves but also in the fungus *Trichoderma reesei*, several mammalian cultured cells and *Spodoptera frugiperda* insect cells (Torrent et al., 2009). The induced protein bodies facilitate stable accumulation of proteins in an encapsulated compartment, thereby protecting the recombinant proteins from degradation by the host cells and reducing the undesirable activities of recombinant proteins on the host. The induced protein bodies also retain the high‐density properties, which facilitate their isolation for purification of the recombinant proteins. Artificial protein bodies have also been recently tested in yeast cells as metabolic vesicles for engineering of a metabolic pathway for cis, cis‐muconic acid production to overcome unwanted side reactions, toxic intermediates, drain of intermediates out of the cell, and long diffusion distances (Reifenrath et al., 2020). Production of cis, cis‐muconic acid from 3‐dehydroshikimate requires three enzymes: 3‐dehydroshikimate dehydratase, protocatechuic acid decarboxylase and catechol dioxygenase. Zera fusions with the three enzymes induce the formation of metabolic vesicles and the incorporation of enzymes based on fluorescence microscopy and cell fractionation techniques. By co‐expressing them in a 3‐dehydroshikimate overproduction yeast strain, activities of the enzymes and functionality of the compartmentalized pathway for production of cis, cis‐muconic acid in the artificially induced protein bodies were successfully demonstrated in fermentation experiments (Reifenrath et al., 2020). Therefore, the cross‐kingdom conservation of protein body formation and the remarkable properties of these specialized ERDVs should make them highly useful in the manufacture of recombinant proteins and metabolites.

## SUMMARY AND PERSPECTIVE

Since protein bodies were first reported as the sites of storage proteins a half century ago (Duvick, 1961), other types of specialized ERDVs including PACs, PPVs (ricinosomes) and ER bodies have been discovered from different plants. Despite the diversity in their morphology, tissue specificity and cargo proteins, these specialized ERDVs share the common features of *de novo* origin from the ER and Golgi‐independent trafficking. It is also apparent that these specialized ERDVs function not only as organelles for processing and storage of massive levels of seed proteins to avoid degradation and ER stress, but also as a platform for signal‐triggered activation and release of enzymes for execution of PCD and defense. Artificially induced ERDVs have been exploited in biotechnology for production of recombinant proteins and metabolic engineering not only in plants but also in non‐plant organisms. Despite these significant advances, important questions remain about the evolutionary relationship, functional diversity, and mechanisms of biogenesis of specialized ERDVs in plants. First, the types of specialized ERDVs that have been characterized so far in plants are primarily defined by the cargo proteins that have been identified, which are very limited. Recent proteomic analysis of natural and artificially induced protein bodies has revealed a large number of non‐zein proteins in protein bodies. In *Arabidopsis*, NAI2‐interacting NAIP2 and NAIP3 are associated not only with ER bodies, which accumulate β‐glucosidases with myrosinase activity, but also with other novel ERDVs with unknown cargos. Therefore, it is unclear about the full fleet of specialized ERDVs and associated cargo proteins in plant cells. Second, the shared ER origin and the Golgi‐independent trafficking could point to the possibility that at least some of these specialized ERDVs are evolutionarily related. Through analysis of NAI2‐interacting NAIP proteins in *Arabidopsis*, we have recently provided evidence that the ER bodies did not originate *de novo* in Brassicaceae plants. More likely they have evolved from a pre‐existing family of ER‐derived structures present in all plants (Wang et al., 2019a). An important question is whether this pre‐existing family of ERDVs, from which the ER bodies have evolved, could be those specialized ERDVs that have already been identified and characterized, such as protein bodies, PACs or PPVs. Third, like any vesicles, biogenesis and trafficking of specialized plant ERDVs are likely to be highly complex, involving distinct machineries for cargo protein recruitment, ER membrane budding, vesicle fission and transport. There is currently little information available about the critical factors that are directly involved in the processes important for the biogenesis and trafficking of specialized ERVs in plants. NAI2 and NAI2‐interacting NAIP2 proteins are directly associated with and required for the formation of specialized ERDVs in plants and are likely to be critical components of the machineries for the biogenesis of specialized ERDVs. However, the exact roles of these proteins in cargo protein recruitment and ERDV biogenesis are still unclear. Given their broad and important biological functions, further understanding of the diversity, function, biogenesis and evolution of specialized ERDVs could provide important new insights into a broad spectrum of biological processes important to plants.

## CONFLICTS OF INTEREST

The authors declare they have no conflicts of interest associated with this work.

## AUTHOR CONTRIBUTIONS

X.L. (for Xie Li) and Z.C. wrote the article and prepared the figures. X.L. (for Xigfeng Li), B. F., and C.Z. cowrote and edited the article. All authors reviewed and approved of the manuscript.
